# Good Genes and Sexual Selection in Dung Beetles (*Onthophagus taurus*): Genetic Variance in Egg-to-Adult and Adult Viability

**DOI:** 10.1371/journal.pone.0016233

**Published:** 2011-01-18

**Authors:** Francisco Garcia-Gonzalez, Leigh W. Simmons

**Affiliations:** Centre for Evolutionary Biology, School of Animal Biology, The University of Western Australia, Nedlands, Australia; Aarhus University, Denmark

## Abstract

Whether species exhibit significant heritable variation in fitness is central for sexual selection. According to good genes models there must be genetic variation in males leading to variation in offspring fitness if females are to obtain genetic benefits from exercising mate preferences, or by mating multiply. However, sexual selection based on genetic benefits is controversial, and there is limited unambiguous support for the notion that choosy or polyandrous females can increase the chances of producing offspring with high viability. Here we examine the levels of additive genetic variance in two fitness components in the dung beetle *Onthophagus taurus.* We found significant sire effects on egg-to-adult viability and on son, but not daughter, survival to sexual maturity, as well as moderate coefficients of additive variance in these traits. Moreover, we do not find evidence for sexual antagonism influencing genetic variation for fitness. Our results are consistent with good genes sexual selection, and suggest that both pre- and postcopulatory mate choice, and male competition could provide indirect benefits to females.

## Introduction

A central tenet in sexual selection is the existence of heritable variation in fitness [1]. Good genes models of sexual selection predict the evolution of female mate choice based on preferences for male traits associated with additive genetic variance in fitness [2]–[4]. There is consensus that, overall, fitness traits exhibit low but non-negligible additive genetic variance [5]–[8]. Importantly, the low heritabilities usually exhibited by fitness components can be explained by high levels of residual variation, rather than by low levels of additive genetic variation [8]. The strength of the association between secondary sexual characters and offspring survival suggests that viability selection may render small but significant increases in fitness [9], but some studies suggest otherwise (e.g., [10]), and the subject of indirect selection on female mate choice or mating behaviour remains a contentious issue in the study of sexual selection [11].

Here we test whether there is significant additive genetic variation in two important components of fitness (egg-to-adult and adult viability) in the dung beetle *Onthophagus taurus*, an increasingly popular model species for the study of precopulatory and postcopulatory sexual selection [12]–[18]. Extensive research on this species converges on a picture of traditional sexual selection based on indirect benefits. Research on this species using a range of experimental tools has so far yielded results that typically meet the requirements for good genes explanations, such as condition dependence of sexually selected traits and genetic correlations between preference and trait ([13], [14], [19] and see Discussion). However, the plausibility of traditional sexual selection based on indirect benefits hinges on the existence of significant contributions by sires to offspring fitness. We have performed a quantitative genetic analysis of egg-to-adult viability and adult survival to sexual maturity, in sons and daughters. We found significant levels of additive genetic variance in offspring fitness, supporting a picture of good genes sexual selection in this species.

## Materials and Methods

### Breeding Design

We used beetles collected in February 2008 from fresh cattle dung in Margaret River, south-west Western Australia, to provide the parental generation for a standard nested paternal full-sib/half-sib breeding design [6], [20]. Wild-collected beetles were kept in mixed sex cultures for 10 days with unlimited access to fresh dung. 250 females were then allowed to construct brood balls for 8 days in individual PVC breeding chambers (further details in [21]) filled with moist sand and topped with 250 ml of fresh cow dung. Each brood ball consists of a chamber containing a single egg surrounded by packed dung that provides the resources for offspring development from hatching to adult emergence. Broods were collected and then buried in sand and incubated at 28±2°C (12 h∶12 h light:dark cycle) until adult emergence. Adults were sexed on emergence, and sexes were maintained separately. Male and female offspring for each of 70 of the initial 250 families were kept in separate boxes, while the offspring of the remaining families were combined by sex and kept in 30-L buckets, three quarters filled with moist sand and topped with 1 L of fresh dung. Sires were extracted from 44 families (one sire per family), while virgin females were randomly extracted from the pool of combined families. Each sire was then housed with 3 dams for 1 week in small plastic containers (7×7×5 cm) half-filled with moist sand and topped with fresh dung. Dams were then established individually, without the sires, in PVC breeding chambers as described above. Non-genetic environmental effects were minimized as the dung provided was homogenised across all dams so that every dam received the same quality and quantity of resources to construct the broods (see below for further discussion on environmental effects). Breeding chambers were sieved for broods after 10 days. Broods were incubated as described above until adult emergence. Male and female offspring were kept in separated 1L plastic boxes, three-quarters filled with moist sand, and topped with ad libitum fresh homogenised dung. All means are presented ± 1SE.

### Egg-to-adult viability and adult survival from emergence to sexual maturity

Given that each individual brood ball contains a single offspring, we were able to measure egg-to-adult viability by monitoring adult emergence from the broods. Egg-to-adult viability can be affected by mortality during embryonic, larval or pupal stages and its transitions. Emergence of adults occurred over the period of two weeks. On emergence beetles undergo a period of maturation feeding, during which males mature their testes and begin to produce sperm, and females mature their ovaries and develop eggs [22]. We monitored survival for 10 days post emergence, or adult survival to sexual maturity. The proportion of emerged adult offspring was independent of the number of brood balls across dam families (r_s_ = 0.14, p = 0.18, n = 102 dam families), and the proportion of offspring surviving was independent of the number of emerged offspring (female offspring r_s_ = 0.06, p = 0.54, n = 98 dam families; male offspring r_s_ = 0.03, p = 0.79, n = 97 dam families). Thus, our fitness measures are unlikely to be influenced by differences among dams in the numbers of broods they produced.

### Sample sizes and quantitative genetic analysis

Sample sizes were reduced from 44 to 36 sires due to low productivity across dam families for 8 sires. Most of the families from these sires were characterized by zero brood production, mostly attributable to infertile matings, whose rates can be appreciable in insect species [23]. Egg-to-adult viability measures were taken from the offspring of 102 dam families distributed across the 36 sires (mean number of dams per sire  = 2.83±0.06, range 2–3; number of broods per dam  = 18.22±0.66, range 2–32; total number of broods  = 1858). The number of dam families for the adult viability data was slightly smaller because a few families failed to produce any adult beetles. Thus, we obtained data for the analysis of son's and daughter's viability for 98 and 97 dam families respectively, distributed across the 36 sires (son's viability: 2.75±0.08 dams per sire, range 1–3; number of sons per dam  = 7.29±0.31, range 1–16, total number of sons  = 714; daughter's viability: 2.70±0.10 dams per sire, range 1–3; number of daughters per dam  = 6.72±0.32, range 1–16, total number of daughters  = 652).

Egg-to-adult viability and adult viability were treated as threshold traits with two values: 0 for non-viable offspring and 1 for viable offspring [20], [24], under the assumption that the variation in viability reflects some underlying continuously distributed character/s that determine liability to survive, which results from the additive action of alleles at several loci [6], [20]. For hypothesis testing (significance of sire and dam effects) we performed Anova III mixed-model nested analyses with dams nested within sires as a random factor in STATISTICA 8.0 [25]. We used Satterthwaite's method of denominator synthesis to account for unequal sample sizes of offspring [26]. Variance component estimation and subsequent analysis of genetic variation was conducted using restricted maximum likelihood (REML) in S-Plus 7.0 [24], using the code for threshold traits generously provided by D. A. Roff. Narrow-sense heritabilities due to sires, heritabilities due to dams, and the genotypic estimate of heritability were calculated on the 0, 1 scale following Roff [27]. The standard errors of the heritabilities were calculated by jacknifing across paternal half-sibling families, and sire and dam heritabilities compared as described in Roff [27]. Coefficients of phenotypic, residual and additive genetic variation (CV_P_, CV_R_ & CV_A_, respectively) were calculated following Houle [8], with additive genetic variation being four times the sire variance component [6]. Genetic correlations between egg-to-adult viability and adult viability were calculated using sire family means, as it is likely that maternal effects, on top of common environmental effects and dominance variance, influence dam estimates in this system [28], [29]. Confidence limits (95%) for the genetic correlations were calculated by bootstrapping using PopTools 3.1.1 [30].

## Results

Egg-to-adult viability and adult male survival exhibited significant additive genetic variance due to sires, but adult female survival did not (Table 1). As expected, due to the known influence of maternal effects in *O. taurus*, dam effects, which also include genetic and common environmental effects, as well as potential dominance variance, were significant for all traits.

**Table 1:** Mixed model nested analyses of variance for egg-to-adult viability and adult male and female viability.

Trait	Source of variance	df	F value	P value
Egg-to-adult viability	Sire1a	35	1.62	0.044
	Dam (Sire)	66	1.91	≪0.001
	Residual	1756		
				
Adult male viability	Sire1b	35	1.85	0.017
	Dam (Sire)	62	2.21	≪0.001
	Residual	616		
				
Adult female viability	Sire1c	35	1.30	0.18
	Dam (Sire)	61	2.22	≪0.001
	Residual	555		

1 Satterthwaite's approximation of the error term to account for unequal sample sizes of offspring within sire (denominator's degrees of freedom, ^a^: 70.32, ^b^: 64.24, ^c^: 61.89).

The coefficients of additive genetic variation (CV_A_) were sizeable for all the traits (Table 2). Coefficients of residual and phenotypic variation were, however, also high, rendering low heritabilities. The genotypic estimates (mean of sire and dam estimates) are significant for all traits, and the sire estimates are not significantly smaller than the dam estimates (Table 2). However, we caution against the use of genotypic estimate due to the likely action of maternal effects inflating dam estimates.

**Table 2:** Descriptive phenotypic and genetic statistics for egg-to-adult viability and adult male and female viability.

Trait	Mean	V_A_	V_P_	h^2^_sire_ (SE)	h^2^_dam_ (SE)	(p_Sire-Dam_)	h^2^_gen._ (SE)	CV_A_	CV_P_	CV_R_
Egg-to-adult viability	0.73	0.012	0.195	0.11 (0.11)	0.29 (0.13)	0.397	0.20 (0.06)	14.73	60.18	58.35
Adult male viability	0.69	0.067	0.218	0.52 (0.35)	0.89 (0.24)	0.505	0.70 (0.16)	37.59	68.08	56.77
Adult female viability	0.67	0.027	0.223	0.20 (0.26)	1.05 (0.33)	0.134	0.63 (0.13)	24.55	70.23	65.80

Mean is the mean proportion calculated as outlined by Roff ([20]; page 57). P-values for the difference between the sire and dam estimate following a t-test of paired sire and dam jackknife pseudovalues (p_Sire-Dam_) are shown [27].

Genetic correlations (sire family means) between egg-to-adult viability and adult viability were not significantly different from zero (males r_g_ = −0.16, p = 0.35, CL -0.44/0.18, n = 36; females r_g_ = 0.013, p = 0.94, CL -0.19/0.24, n = 36) suggesting that these components of fitness are likely to be independent. The genetic correlation between son and daughter adult viability was positive, but marginally non significant (r_g_ = 0.31, p = 0.064, CL -0.14/0.69, n = 36).

## Discussion

We have found significant sire effects on egg-to-adult viability and son's pre-reproductive survival, as well as moderate to high coefficients of additive genetic variation for these traits, and for daughter's pre-reproductive survival in *O. taurus*. These results indicate that there is genotypic variation among males that translates into variation in offspring viability. This variation provides the raw material for the accruement of indirect benefits resulting from female choice.

Genetic benefits in *O. taurus* can arise by both precopulatory and postcopulatory sexual selection acting on traits that are genetically linked to condition. Models of sexual selection based on indirect benefits require condition dependence for sexual traits if they are to be reliable signals for female choice [31]. In *O. taurus*, traits targeted by precopulatory female choice such as courtship rate, or postcopulatory sexual selection such as testis weight, ejaculate volume, and sperm length, exhibit significant additive genetic variation and depend on condition, which is itself heritable [13], [19]. There is a preference for males with high courtship rates [13], males of high genetically determined condition have larger testes and shorter sperm [19], and short sperm have been found to give males an edge in competitive fertilization success [16]. Cryptic female choice on sperm length is supported by a genetic correlation between spermathecal size and sperm length congruent with the sexy sperm model, and by an interaction between these two traits in determining paternity success [14], [16]. The genetic relationship between sperm length and male condition suggests that the good sperm model, which requires genetic integration between competitive fertilization success in males and offspring viability [32] is also plausible. Recent findings from an experimental evolution approach add further support for good genes. Females from monogamous lineages exhibited higher offspring condition when mating polyandrously than when mating monogamously [21], indicating that polyandry may serve females to increase offspring genetic quality through the facilitation of sperm competition: most fertilizations will be achieved by competitive males who are expected to sire offspring of higher condition due to the genetic association between male condition and ejaculate traits that determine fertilization success. Importantly, there is evidence that males in better condition sire offspring of higher condition and greater viability [17]. Collectively, our results combined with previous research suggest that both precopulatory and postcopulatory mate choice and male competition could provide indirect benefits to choosy or polyandrous females. In the absence of evidence for the view that the interaction between males and females in this system is driven by sexual conflict (see below and [21]), a picture of good genes as explanation for the evolution of mating preferences and mating rates in *O. taurus* seems most likely. We have measured two important components of fitness, egg-to-adult and adult viability, but we note that using fitness components as proxies for fitness can be problematic if there are trade-offs between measured and unmeasured fitness components [33].

Life-history traits typically exhibit lower heritabilities than traits with tenuous connection to fitness, but the former generally exhibit higher coefficients of additive genetic variation (CV_A_) [8]. CV_A_ for egg-to-adult viability in *O. taurus* (14.73) is in line with coefficients reported for life-history traits in *Drosophila* [8] and with CV_A_s for egg-to-adult survival in other species (e.g., [34]), while CV_A_ for early adult viabilities are much higher (37.59 for male, and 24.55 for female offspring), potentially indicating high evolvability for these traits. As expected if fitness traits are determined by a large number of loci, residual variance is high for all the three traits examined in this study [8]. Heritabilities are consequently low, in agreement with theoretical expectations.

We have found that sire effects on son survival were stronger than for that of daughters. Interestingly, Simmons and Kotiaho [19] found patterns indicative of Y-linked inheritance in condition-dependent traits, and importantly, a recent independent study [17] has confirmed a link between male condition and son's but not daughter's condition (see below). There is increasing evidence that the genetic correlation for fitness between the sexes is often negative. This is the case in *Drosophila melanogaster* [35]–[37], the red deer [38], the cricket *Allonemobius socius* [39] and probably the seed beetle *Callosobruchus maculatus* [40]. Such patterns underscore the existence of intralocus sexual conflict negating or minimizing potential indirect genetic benefits of sexual selection. However, our results in *O. taurus* do not support the notion that genetic variation for fitness is affected by sexual antagonism: the genetic correlation for adult viability between males and females is marginally non-significant but positive instead of negative (r_g_ = 0.31, p = 0.064, CL -0.14/0.69, n = 36). Sex ratio at adult emergence or at sexual maturity did not differ from 0.5 across dam or sire families (all means  = 0.53, all p's from t-tests >0.05). Nonetheless, the existence of a negative correlation for fitness between the sexes in *O. taurus* cannot be ruled out completely with our current data; the analysis of the covariance between further fitness components (e.g., lifespan, reproductive success, etc.) across the sexes would provide a more complete test of sexual antagonism in total fitness.

One of the biggest challenges facing evolutionary genetics is the separation of purely direct genetic effects on offspring performance from female differential allocation or maternal/paternal effects [41]. Dung beetles are a clear example where these influences merge, because of strong maternal and paternal effects on offspring size and development that arise from parental provisioning during the construction of brood balls [18], [28], [29]. In this study paternal effects arising from paternal care were controlled for, and potential differential allocation effects are likely to be of lesser importance than genetic effects: Simmons [17] recently conducted a maternal split clutch design in which females were mated doubly with both high and low condition males (one of the males irradiated to render unviable the embryos he sired), and analyzed survival from hatching to sexual maturity. This study, which effectively controlled for maternal effects, found significant covariation between the condition of a male and his surviving sons but not between male-daughter condition [17]. Such results mirror our findings of significant sire effects on sons' but not on daughters' viability. Thus, genetic effects seem to have a preponderant role.

In sum, the results in this study, together with previous research on *O. taurus*, support the notion that females can obtain genetic benefits in the form of increased offspring viability as a result of female mate choice and polyandrous behaviour.
